# Role of the calcium‐sensing receptor in regulating vascular function

**DOI:** 10.1002/ccs3.70004

**Published:** 2025-02-05

**Authors:** Anthony P. Albert, Harry Z.E. Greenberg

**Affiliations:** ^1^ Vascular Biology Section Cardiovascular & Genomics Research Institute St. George's, University of London London UK; ^2^ Norwich Medical School University of East Anglia Norfolk UK

**Keywords:** calcification, calcium‐sensing receptor, contractility, proliferation, vascular

## Abstract

Functional expression of the calcium‐sensing receptor (CaSR) in calcitropic tissues, for example, parathyroid glands and kidneys, is important for maintaining Ca^2+^ homeostasis. It is also established that the CaSR is present in tissues previously thought to be noncalcitropic and this review discusses the role of the CaSR in vascular function, focusing mainly on contractility but also outlining its role in cell proliferation and calcification. Stimulation of the CaSR by extracellular Ca^2+^ concentration ([Ca^2+^]_o_) on perivascular sensory nerves and vascular endothelial cells is associated with vasodilatation through the release of vasoactive substances and stimulation of IK_Ca_ channels and nitric oxide synthesis, respectively, which mediate endothelium‐derived hyperpolarizations and activation of BK_Ca_ channels and K_ATP_ channels in vascular smooth muscle cells (VSMCs). CaSR‐induced vasoconstrictions are mediated by the CaSR expressed in VSMCs, which are coupled to the G_q/11_ protein‐coupled pathway. In addition, the CaSR expressed on VSMCs also regulates proliferation and calcification. Consequently, the CaSR has been implicated in regulating systemic and pulmonary blood pressure and calcimimetics and calcilytics are potential therapeutic targets for cardiovascular diseases, such as hypertension, pulmonary artery hypertension, and atherosclerosis.

## Graphical abstract

Representation of the role of the CaSR in the vasculature shows that a rise in extracellular Ca^2+^ concentration ([Ca^2+^]_o_) stimulates the CaSR expressed at the plasma membrane of perivascular sensory nerves, VECs, and VSMCs, which leads to the release of vasodilator substances (e.g., calcitonin‐related gene‐related peptide (CGRP), substance P (Sub P), and lipid substances), activation of vasodilatation via nitric oxide (NO)‐ and endothelium‐derived hyperpolarization (EDH)‐mediated pathways and vasoconstriction, and proliferation and calcification of VSMCs, respectively.

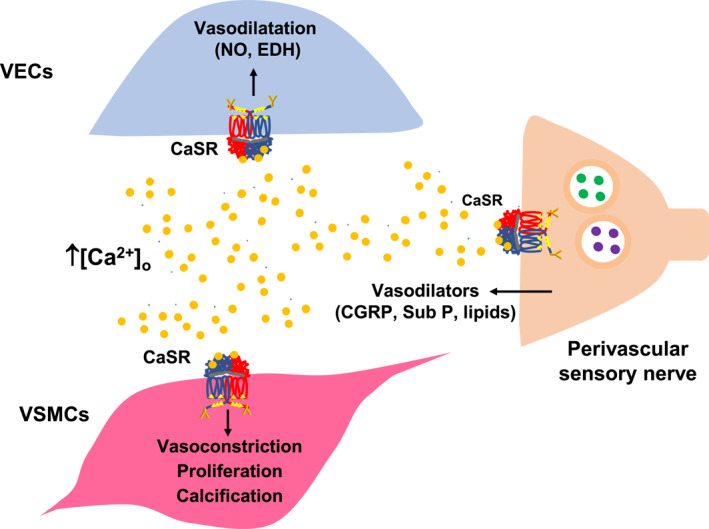

## 1 INTRODUCTION

It is well‐established that the regulation of the calcium‐sensing receptor (CaSR) by extracellular Ca^2+^ concentration ([Ca^2+^]_o_) is pivotal for maintaining total blood plasma calcium levels within a range of 2.1–2.6 mM, with free ionized Ca^2+^ at around 1.2 mM.^1^, ^2^, ^3^, ^4^ These levels are achieved through the functional expression of the CaSR in the parathyroid glands, with a rise in [Ca^2+^]_o_ suppressing synthesis and secretion of parathyroid hormone (PTH) to reduce plasma Ca^2+^ levels through actions on intestinal Ca^2+^ absorption, renal Ca^2+^ excretion, and bone remodeling. In addition, the CaSR is present in renal tubules where they regulate Ca^2+^ reabsorption. Dysfunction of the CaSR and associated cell signaling pathways leads to calcitropic diseases, with loss of function mutations causing high plasma Ca^2+^ levels, for example, familial hypocalciuric hypercalcemia and gain of function mutations producing low Ca^2+^ levels, for example, autosomal dominant hypocalcaemia. In addition, primary and secondary hyperparathyroidisms are associated with changes in CaSR expression levels.^3^, ^4^ Thus, the CaSR is a therapeutic target with the clinically approved calcimimetics or positive allosteric modulators (PAMs), cinacalcet, etelcalcetide, and upacicalcet, which are used to increase the sensitivity of the CaSR to [Ca^2+^]_o_ in the parathyroid glands to treat secondary hyperparathyroidism.^3^, ^4^

The CaSR is also present in tissues previously thought to not be involved in plasma Ca^2+^ homeostasis, for example, the nervous system, gastrointestinal tract, and the cardiovascular system.^3^, ^4^ However, recent evidence indicates that the functional expression of the CaSR in previously described noncalcitropic tissues such as the vasculature may indeed also modulate Ca^2+^ homeostasis.^3^, ^5^

Over the past 25 years, there has been growing evidence that the CaSR has important roles in the vasculature and this review focuses on our current understanding of the expression, associated cellular mechanisms, and function of the CaSR in regulating vascular contractility and proliferation and calcification of vascular smooth muscle cells (VSMCs). There are excellent reviews on other various aspects of the CaSR in the cardiovascular system, such as in cardiac tissue and modulation of blood pressure during sepsis (see ^6^, ^7^, ^8^, ^9^, ^10^, ^11^).

## 2 BACKGROUND TO THE CaSR

The CaSR is a member of the Class C family of G‐protein‐coupled receptors, including the metabotropic glutamate receptor and gamma‐aminobutyric acid B receptor, which contain an extensive extracellular structure termed a Venus flytrap domain which mediates the activator and allosteric modulator binding (see recent reviews by ^3^, ^4^, ^12^, ^13^, ^14^, ^15^, ^16^, ^17^ for comprehensive accounts of the general structure, function, and pharmacology of the CaSR and its role in physiology and diseases of calcitropic and other nonvascular calcitropic tissues). A characteristic of the CaSR is that it forms functional homomeric and heteromeric dimers with other Class C receptors. The CaSR is primarily coupled to the activation of the G_q/11_ protein‐coupled pathway leading to phospholipase C (PLC) stimulation, phosphoinositol 4,5‐bisphosphate (PIP_2_) hydrolysis, and generation of diacylglycerol (DAG) and inositol 1,4,5‐trisphosphate (IP_3_) and raising the intracellular Ca^2+^ concentration ([Ca^2+^]_i_). CaSR‐mediated rise in [Ca^2+^]_i_ is also associated with the activation of calmodulin kinase II, stimulation of cytosolic phospholipase A_2_ (cPLA_2_), and the actin‐binding protein filamin A. In addition, the CaSR is coupled with G_i/o_ and G_12/12_ proteins leading to a reduction in cyclic adenosine monophosphate and stimulation of mitrogen‐activated protein kinase (MAP kinase) cascades, RhoA kinase, and PI‐3/PI‐4 kinase pathways.

The CaSR is stimulated by [Ca^2+^]_o_ and other divalent and trivalent cations such as Mg^2+^ and Gd^3+^, polyamines (e.g., spermine), and aminoglycoside antibiotics (e.g., neomycin), and it is proposed that activators of the CaSR may elicit biased agonism.^3^ Moreover, endogenous molecules also act as allosteric modulators of the CaSR, for example, L‐amino acids and phosphate, which bind to and increase the sensitivity of other activators at the CaSR.^17^ The development of allosteric modulators forms the basis for current CaSR pharmacology, with calcimimetics or PAMs (clinically approved cinacalcet, etelcalcetide, and upacicalcet or compounds used for research purposes, e.g., R‐568) increasing the sensitivity to [Ca^2+^]_o_, whereas calcilytics or negative allosteric modulators (NAMs, e.g., clinically trialled compounds AXT914, AFT936, and ronacalert and research compounds NPS‐2143 and Calhex‐231) reduce sensitivity (see Nemeth & Goodman ^13^ for a detailed evaluation of calcimimetics and calcilytics).

## 3 EXPRESSION OF THE CaSR IN THE VASCULATURE

Blood vessels are structured into three main layers: the outer adventitial layer containing perivascular nerves (including sympathetic and perivascular sensory nerves), the medial layer containing VSMCs, and the innermost intimal layer containing vascular endothelial cells (VECs), which “sense” the pressure, flow, and contents of blood as it passes through the lumen. These three main cell types communicate with each other to regulate vascular contractility and control blood pressure and flow.

In the rat, the CaSR has been located on perivascular nerves using immunocytohistochemical (IHC) in mesenteric arteries,^18^, ^19^, ^20^, ^21^, ^22^, ^23^ renal, coronary, and cerebral arteries ^20^, ^24^ and cloned from rat dorsal root ganglion (DRG) neurones.^25^ The CaSR has been identified in VSMCs from gerbil spiral modiolar artery using polymerase chain reaction (PCR),^26^ rat mesenteric artery using western blotting (WB), IHC, and immunocytochemistry (ICC) ^22^, ^23^, ^27^, aorta using PCR and ICC,^28^ and pulmonary artery by PCR, WB, and ICC,^29^ human renal, epigastric, tibial, and internal mammary arteries using PCR, WB, and IHC,^30^, ^31^ mouse aorta by PCR, WB, and IHC,^32^ and rat aorta by WB and IHC.^33^, ^34^ CaSR expression has been described in VECs from the rat and rabbit mesenteric artery using IHC and ICC ^21^, ^22^, ^23^, ^35^, human aortic endothelial cells (HAECs) using PCR, WB, and ICC,^36^ and human umbilical vein endothelial cells (HUVECs) using WB and ICC.^37^

These findings indicate that the CaSR is widely expressed in different vascular beds from many species, which suggest that CaSR‐mediated responses are likely to have a canonical role in the vascular function. The expression of the CaSR in perivascular nerves, VSMCs and VECs, often in the same vascular bed, also suggests that CaSR‐mediated responses are likely to be complex.

## 4 [Ca^2+^]_o_ AND STIMULATION OF THE CaSR AS A POTENTIAL PARACRINE SIGNALING SYSTEM WITHIN BLOOD VESSEL WALLS

Plasma Ca^2+^ levels are highly regulated and maintained within strict limits due to the action of the CaSR at calcitropic tissues but it is recognized that localized [Ca^2+^]_o_ may significantly vary compared to plasma Ca^2+^ levels according to the cell type and function.^2^, ^38^ It is proposed that extracellular sub‐compartments or microenvironments are likely to exist where Ca^2+^ acts as the paracrine signaling system, producing intercellular communication via the CaSR expressed on localized networks of cells.^39^ Moreover, allosteric and orthosteric modulators such as Mg^2+^, phosphate, and L‐amino acids in this localized environment may also sensitize CaSR‐mediated actions.

**FIGURE 1:**
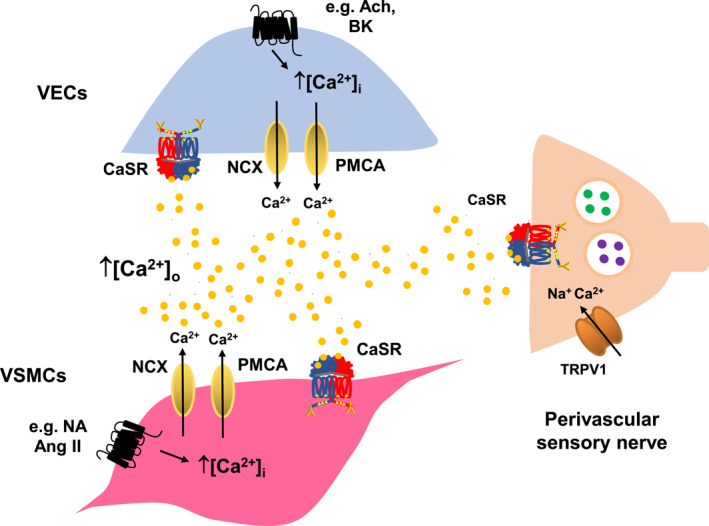
Causes of localized [Ca^2+^]_o_ rise in the vascular tissue. Stimulation of receptors leads to a rise in [Ca^2+^]_i_ in VECs and VSMCs, which is returned to resting levels by the sodium‐calcium exchanger (NCX) and plasma membrane Ca^2+^‐ATPase (PMCA). As a consequence of these mechanisms, localized [Ca^2+^]_o_ rises which can lead to the stimulation of the CaSR functionally expressed on VECs, VSMCs, and perivascular sensory nerves. Ach, acetylcholine; Ang II, angiotensin II; BK, bradykinin; CaSR, calcium‐sensing receptor; NA, noradrenaline; NCX, sodium‐calcium exchanger; PMCA, plasma membrane calcium ATPase; TRPV1, vanilloid transient receptor potential channel 1; VECs, vascular endothelial cells; VSMCs, vascular smooth muscle cells.

Vasoactive substances acting at G_q/11_‐protein‐coupled receptors on VECs and VSMCs stimulate increases in [Ca^2+^]_i_ through the activation of Ca^2+^ release from Ca^2+^ stores and Ca^2+^ influx through Ca^2+^‐permeable channels (e.g., voltage‐gated Ca^2+^ channels (VGCCs) and transient receptor potential channels (TRP)), which regulate vascular contractility through multiple pathways involving nitric oxide (NO), NO production, and release from VECs and regulation of ion channel activities involved in controlling membrane potential and regulation of the Ca^2+^‐CaM‐myosin light chain kinase (myosin light chain kinase) contractile system in VSMCs. In response, Ca^2+^ extrusion mechanisms are activated in VECs and VSMCs, for example, Ca^2+^‐ATPase and Na^+^/Ca^2+^ exchanger, which lower [Ca^2+^]_i_ while increasing localized [Ca^2+^]_o_ that may activate the CaSR expressed on perivascular sensory nerves, VSMCs, and VECs in the blood vessel wall ^32^, ^40^, ^41^ (Figure 1). In this regard, it is worth reflecting that the activation of K^+^ channels in VECs leads to a localized rise in extracellular K^+^ concentration ([K^+^]_o_) to over 10 mM, which acts as an endothelium‐derived relaxing factor to activate Na^+^/K^+^‐ATPase and K_ir_ channels on VSMCs that induces hyperpolarizations and vasorelaxations.^41^, ^42^

As described above, it is possible and likely that localized [Ca^2+^]_o_ increases above plasma levels in the vascular tissue. However, it should be remembered that a rise in [Ca^2+^]_o_ may regulate other mechanisms, apart from the CaSR, that control the vascular function. For example, [Ca^2+^]_o_ is proposed to modulate the K_ir_ channel and Na^+^/K^+^‐ATPase activities which are known to modulate vasorelaxations.^43^ It is therefore important to investigate [Ca^2+^]_o_‐induced changes in the vascular function using pharmacological or molecular approaches for the CaSR.

The work described in this article builds upon the early studies of Ruat et al. ^24^ and the Bukoski group.^18^, ^44^ Ruat et al. ^24^ cloned the CaSR in rat neurones and used IHC studies to show the presence of the CaSR in discrete punctate at nerve terminals but not cell bodies and that the CaSR was present in a dense network of nerves surrounding rat cerebral arteries. Bukoski's group showed using PCR techniques that CaSR mRNA was present in DRG neurones but not in rat mesenteric artery tissues, whereas IHC studies showed that the CaSR protein was present in nerve networks within the adventitial layer of vessels.^18^ Moreover, Bukoski's group showed functional actions of the CaSR on vascular contractility by demonstrating that increasing [Ca^2+^]_o_ from 0 to 0.8 mM augmented the precontracted tone in rat mesenteric arteries, whereas increasing [Ca^2+^]_o_ above 0.8 mM produced concentration‐dependent relaxations with an effective concentration at 50% (EC_50_) of about 2.5 mM [Ca^2+^]_o_ and near complete relaxation at higher concentrations.^18^, ^44^ These findings indicated that the stimulation of the CaSR in the vasculature was likely regulating vascular contractility at normal plasma Ca^2+^ levels.

## 5 CaSR‐MEDIATED REGULATION OF VASCULAR CONTRACTILITY: VASODILATATION OR CONSTRICTION?

Numerous studies have investigated the effect of stimulating the CaSR on vascular contractility, with the majority of these works examining the effect of increasing [Ca^2+^]_o_ on the precontracted tone using wire or pressure myography and organ bath techniques. As might be expected from a varied CaSR expression on different cell types in the vasculature as highlighted above, CaSR‐mediated actions on vascular contractility include both dilatator and constrictor responses.

### 5.1 CaSR‐mediated vasodilatations

In rat, rabbit, and mouse mesenteric arteries, increasing [Ca^2+^]_o_ induced relaxations of the precontracted tone with an EC_50_ of about 2.5 mM, which were inhibited by Calhex‐231.^18^, ^19^, ^20^, ^35^, ^44^, ^45^, ^46^, ^47^, ^48^ Similar [Ca^2+^]_o_‐induced relaxations were observed in rat coronary, renal, and cerebral arteries ^20^ and mouse aorta,^49^ whereas the calcimimetic AMG073 induced relaxations in the rat aorta.^50^, ^51^ In contrast, recent studies reported [Ca^2+^]_o_‐induced relaxations of the precontracted tone with a lower sensitivity to [Ca^2+^]_o_ (EC_50_ about 5 mM) in rat mesenteric arteries which were inhibited by Calhex‐231.^22^, ^23^ The reasons for these different findings in the rat mesenteric artery are unclear, but they may reflect different vasoconstrictors and concentrations used to produce the precontracted tone and different orders of the vessel arcade used.^22^

### 5.2 CaSRs‐mediated vasoconstrictions

In superfused gerbil spiral modiolar artery segments, increasing [Ca^2+^]_o_ from 1 to 10 mM increased the precontracted tone, which was mimicked by neomycin and Gd^3+^.^26^ In rat pulmonary arteries, increasing [Ca^2+^]_o_ from 5 to 12.5 mM augmented the precontracted tone, which was reduced by NPS2390, a mGluR antagonist, and calcilytic.^13,^ ^29^ In a transgenic mouse study, contractions to the alpha_1_‐adrenoceptor agonist phenylephrine (PE) and KCl in aorta and mesenteric arteries were reduced in preparations from smooth muscle‐specific CaSR knockout (KO) mice compared to wild‐type (WT).^32^ In addition, increasing [Ca^2+^]_o_ from 1 to 5 mM contracted PE‐evoked the precontracted tone in aorta segments from WT but induced relaxations in KO vessels. Moreover, this study showed that increasing [Ca^2+^]_o_ produced relaxations of mesenteric arteries which were similar in WT and KO mice. Interestingly, these findings in the mouse aorta are in contrast to the earlier work showing that increasing [Ca^2+^]_o_ induced relaxations of the mouse aorta.^49^ Importantly, KO mice had a small but significant lower systemic blood pressure (decrease of 5% systolic, 9% diastolic, and 7% mean arterial pressure), with cardiac remodeling and bradycardia. Together, this KO study indicated that the CaSR is involved in regulating systemic blood pressure, but this may be due to changes in heart rate, cardiac contractility, and vascular resistance. This transgenic mouse study may also suggest that the CaSR mediates vasoconstrictions through the functional expression on VSMCs in predominantly large arteries like the aorta, whereas CaSR‐mediated vasodilatations presumably through the functional expression on perivascular sensory nerves and VECs may be dominant in more resistance vessels such as mesenteric arteries.

### 5.3 CaSR‐mediated biphasic responses

In rat subcutaneous arteries, increasing [Ca^2+^]_o_ from 0.5 to 3 mM induced small constrictions of the resting myogenic activity, whereas increasing [Ca^2+^]_o_ above 3 mM induced relaxations, with these biphasic responses mimicked by neomycin and Mg^2+^.^27^ Interestingly, on the abolishment of [Ca^2+^]_o_‐induced relaxations in rabbit and rat mesenteric arteries by the removal of a functional endothelium or inhibiting both IK_Ca_ channels and NO production ^35^ or inhibiting both calcitonin‐related gene‐related peptide (CGRP) and neurokinin 1 (NK1) receptors, respectively,^23^ increasing [Ca^2+^]_o_ now produced pronounced augmentation of the precontracted tone. These findings further suggest that CaSR‐mediated changes in vascular contractility may be a balance between relaxation and constriction responses, with dominance varying in different vascular preparations.

## 6 CaSR‐MEDIATED CELLULAR SIGNALING MECHANISMS INVOLVED IN REGULATING VASCULAR CONTRACTILITY

**FIGURE 2:**
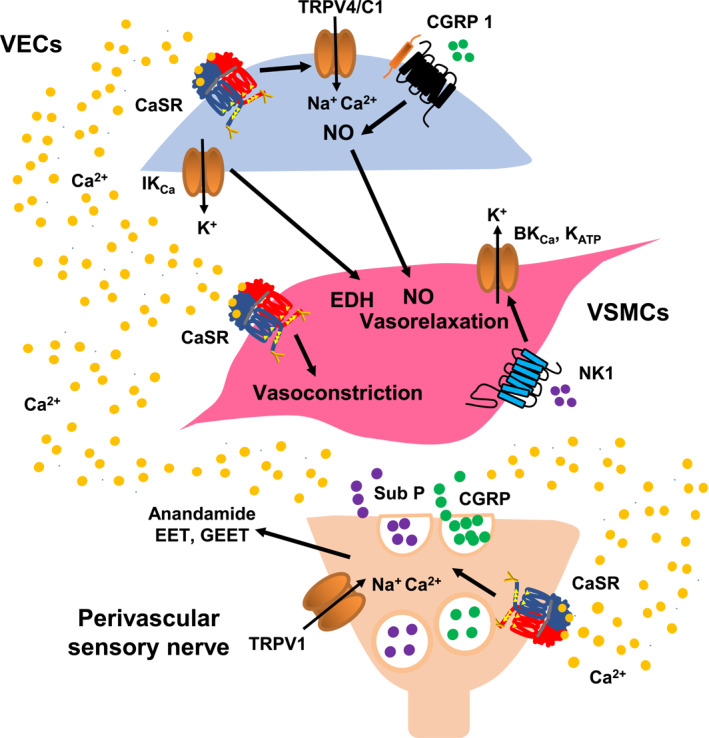
Summary of proposed mechanisms involved in regulating CaSR‐mediated changes in vascular contractility. Stimulation of the CaSR on perivascular nerves induces the release of vasoactive substances, which act at VECs (e.g., CGRP) or directly on VSMCs (e.g., anandamide, EET, GEET, and Sub P) to lead to vasodilatation. Stimulation of the CaSR on VECs and VSMCs induces vasodilatation and vasoconstriction, respectively. It is proposed that vasodilatations mediated by VECs involve TRPV4/C1 channel‐mediated NO production and release and activation of IK_Ca_ channels, whereas the activation of different K^+^ channel subtypes (e.g., BK_Ca_, SK_Ca_, and K_ATP_) channels mediated vasodilatations in VSMCs. Stimulation of the CaSR on VSMCs induces vasoconstriction through activating the G_q/11_ protein‐coupled pathway. It is likely that CaSR‐mediated effects on vascular contractility are a balance between these multiple vasodilatation and vasoconstriction pathways. BK_Ca_, large conductance calcium‐activated potassium channel; CaSR, calcium‐sensing receptor; CGRP, calcitonin gene‐related peptide; CGRP 1, calcitonin gene‐related peptide 1 receptor; EDH, endothelium‐derived hyperpolarization; EET, epoxyeicosatrienoic acids; GEET, glycerated EET; IK_Ca_, large conductance calcium‐activated potassium channel; K_ATP_, ATP‐activated potassium channel; NK1, neurokinin 1 receptor; NO, nitric oxide; Sub P, substance P; TRPV1, vanilloid transient receptor potential channel 1; TRPV4/C1, heteromeric vanilloid transient receptor potential 4 and canonical transient receptor potential 1 channel; VECs, vascular endothelial cells; VSMCs, vascular smooth muscle cells.

Given that the regulation of the vascular tone through the relaxation and contraction of VSMCs is governed by interactions between vasoactive substances released from perivascular nerves, VECs, perivascular fat, and immune cells, and also actions of bloodborne hormones and other molecules, it is not surprising that studies investigating the role of the functional expression of the CaSR on perivascular sensory nerves, VECs, and VSMCs in regulating vascular contractility have identified diverse and sometimes contradictory cellular mechanisms. These findings are discussed below and summarized in Figure 2.

### 6.1 Perivascular sensory nerve‐dependent actions

There is increasing consensus that the stimulation of the CaSR on perivascular sensory nerves provide a significant contribution to [Ca^2+^]_o_‐induced relaxations in rat mesenteric arteries, although there are differing proposals for signaling pathways involved.

Initial evidence indicated that denervation of perivascular nerves with acute phenol pretreatment reduced [Ca^2+^]_o_‐induced vasorelaxations of the precontracted tone in rat mesenteric arteries.^18^, ^20^, ^52^, ^53^ In addition, the electrical field stimulation of perivascular nerves, in the presence of guanethidine to block the release of noradrenaline from sympathetic nerves, relaxed the precontracted tone.^20^, ^53^ These findings have been substantiated by showing that the chronic pretreatment of vessels with capsaicin, which acts at TRPV1 to produce desensitization of perivascular sensory nerves and reduced density of CGRP and CaSR neuronal staining,^19^ also reduced [Ca^2+^]_o_‐induced vasorelaxations ^19^, ^23^, ^48^. Electrical field stimulation‐mediated vasorelaxations in renal, coronary, and cerebral arteries also indicate that perivascular nerves may be involved in CaSR‐induced responses in other vasculature beds.^20^

The above studies agree that the stimulation of the CaSR in perivascular sensory nerves in rat mesenteric arteries leads to signaling pathways that ultimately cause the activation of K^+^ channels, hyperpolarization, and relaxation of VSMCs. High KCl concentration‐induced contractions, which clamp VSMCs at a depolarizing membrane potential leading to the activation of VGCCs, Ca^2+^ influx, and contraction, are not relaxed by increasing [Ca^2+^]_o_, indicating that hyperpolarization is essential.^22^, ^52^ Moreover, [Ca^2+^]_o_‐induced vasorelaxations are completely inhibited by tetraethylammonium, a nonselective voltage‐gated K^+^ channel blocker, and partially reduced by BK_Ca_ channel blockers.^22^, ^45^, ^52^ The partial reduction of [Ca^2+^]_o_‐induced vasorelaxations following the removal of a functional endothelium also indicates that CaSR‐mediated perivascular sensory nerve pathways may involve endothelium‐coupled processes.^22^, ^46^

Studies by the Bukoski group indicated that [Ca^2+^]_o_‐induced vasorelaxations were not affected by inhibitors of CGRP receptors (CGRP 8–37), NK1, and neurokinin 2 receptors (Spantide II and SR140333, and SR48968 respectively) or eNOS (NG‐nitro‐L‐arginine methyl ester [L‐NAME]).^18^ However, [Ca^2+^]_o_‐induced vasorelaxations were reduced by inhibitors of cytochrome p450 (miconazole), PLA_2_ (quinacrine, AACOCF_3_), cannabinoid receptor 1 (CB_1_, SR141716A), and DAG lipase (RHC‐80267)^18^, ^45^, ^46^ and enhanced by pefabloc that prevents anandamide breakdown.^45^ These findings proposed that the stimulation of the CaSR on perivascular sensory nerves leads to the production and release of cytochrome P_450_ and cPLA_2_ metabolites such as glycerated epoxyeicosatrienoic acids which act at BK_Ca_ channels on VSMCs to produce hyperpolarization and vasorelaxation.^46^ In WT and CB_1_ KO mice, [Ca^2+^]_o_‐induced vasorelaxations were both reduced by SR141716 A, suggesting that CaSRs‐mediated anandamide production and release from perivascular sensory nerves may act via a nonclassical CB_1_‐like receptor on VSMCs to activate BK_Ca_ channels.^46^, ^54^

Recent studies have also proposed different signaling cascades that couple the stimulation of the CaSR in perivascular sensory nerves to vasorelaxations in rat mesenteric arteries, with [Ca^2+^]_o_‐induced relaxations reduced by L‐NAME, CGRP 8–37 BIB‐4096 (another CGRP receptor blocker), L733,060 (NK1 receptor blocker), and PNU37883 (ATP‐activated postassium channel [K_ATP_] blocker).^22^, ^23^ In addition, there was little effect of indomethacin (cyclooxygenase [COX] inhibitor), CAY10441 (IP receptor), SR141716A, charybdotoxin (CbTX, IK_Ca_, and BK_Ca_ channel blocker), BaCl_2_ (K_ir_ channel blocker), and linopidine (Kv7 channel blocker) on [Ca^2+^]_o_‐induced relaxations. Interestingly, [Ca^2+^]_o_‐induced vasorelaxations were completely abolished by the co‐application of CGRP 8–37 and L733,060, and the individual inhibitory actions of CGRP 8–37 and iberiotoxin (BK_Ca_ channel blocker) were prevented by the removal of a functional endothelium, whereas the actions of PNU37883 were unaffected.^22^, ^23^ These results proposed that the stimulation of the CaSR on perivascular sensory nerves induced vasorelaxations through 1) CGRP release and an endothelium‐dependent pathway involving NO production and activation of BK_Ca_ channels in VSMCs and 2) substance P release and an endothelium‐independent pathway involving the activation of K_ATP_ channels in VSMCs.

It is unclear why there are differences between the proposed cellular signaling pathways mediating vasorelaxations induced by the stimulation of the CaSR on perivascular sensory nerves in rat mesenteric arteries; it may be due to different orders of vessels used, different vasoconstrictors and concentrations used to produce the precontracted tone, and/or involvement of other receptors that may sense Ca^2+^, for example, the GPRC6A receptor.^55^, ^56^ It may also be due to nonselectivity of agents used in these pharmacological studies, for example, cytochrome p450 inhibitor miconazole is also known to block K^+^ channels and VGCCs.^57^, ^58^ The differing effect of CGRP and NK1 receptor blockers on [Ca^2+^]_o_‐induced vasorelaxations is more difficult to explain. Further work is required to more critically evaluate the cellular pathways involved in mediating vasorelaxations induced by the stimulation of the CaSR on perivascular nerves in rat mesenteric arteries, using more molecular approaches alongside pharmacological agents.

### 6.2 Endothelium‐dependent actions

Stimulation of the CaSR in VECs is also proposed to contribute to [Ca^2+^]_o_‐induced relaxations in rat mesenteric arteries, with the calcimimetic calindol inducing hyperpolarizations of VSMCs which were prevented by Calhex‐231, removal of a functional endothelium, IK_Ca_ channel blockers TRAM‐39 and TRAM‐34, and a combination of the K_ir_ channel blocker BaCl_2_ and Na^+^/K^+^‐ATPase inhibitor ouabain.^21^, ^41^ These results led to the proposal that the stimulation of the CaSR and activation of IK_Ca_ channels in VECs induce a localized rise in [K^+^]_o_ which activates K_ir_ channels and Na^+^/K^+^‐ATPase on VSMCs to produce hyperpolarizations and vasorelaxations.^41^ Intriguingly, this work suggested that the vasoconstrictor‐induced BK_Ca_ channel activity in VSMCs may lead to a localized interstitial “K^+^ cloud” which prevents CaSR‐mediated IK_Ca_ channel‐induced hyperpolarizations from being observed, a potential reason why an action for the CaSR in VECs had not previously been proposed in this preparation.

In rat mesenteric arteries, Dora et al. ^40^ also proposed another interesting role for the CaSR whereby an acute increase in [Ca^2+^]_o_ from 1 to 3 mM evoked vasorelaxations of the precontracted tone which were blocked by the removal of a functional endothelium and TRAM‐34 but upon chronic pretreatment with 2.5 mM [Ca^2+^]_o_, IK_Ca_ channel responses were reduced. This CaSR‐mediated IK_Ca_ channel inhibition could be restored by incubating vessels with 1 mM [Ca^2+^]_o_ or by vasoconstrictor‐mediated activation of VGCCs to lower interstitial Ca^2+^ levels and thus prevent the CaSR from being activated.

In HAECs, the calcimimetic spermine, but not [Ca^2+^]_o_, induced NO production that was blocked by L‐NAME and was proposed to be due to the stimulation of a CaSR splice variant, which lacks exon 5 and is insensitive to [Ca^2+^]_o_.^36^ An alternative CaSR splice variant lacking exon 5, exhibiting with poor activation by [Ca^2+^]_o_, has previosuly been described in keratinocytes.^59^ In rat aorta, AMG‐073‐mediated, but not neomycin‐ or Gd^3+^‐induced relaxations were inhibited by a combination of L‐NAME and indomethacin which indicated the involvement of an endothelium‐dependent pathway in CaSR‐mediated relaxations, although it was not confirmed whether the CaSR was present on VECs or another cell type such as perivascular sensory nerves.^28^, ^50^, ^51^ In the mouse aorta, [Ca^2+^]_o_‐induced relaxations were abolished by the removal of a functional endothelium and pretreatment with L‐NAME, suggesting that the stimulation of the CaSR on VECs is important to these responses.^49^ In mesenteric arteries from eNOS KO mice, [Ca^2+^]_o_‐induced relaxations of the precontracted tone were greatly reduced compared to WT, whereas [Ca^2+^]_o_‐induced relaxations were unaffected in vessels from nNOS KO mice.^47^

In rabbit mesenteric arteries, [Ca^2+^]_o_‐induced relaxations were completely abolished by the removal of a functional endothelium and combination of L‐NAME with CbTx (BK_Ca_ channel and IK_Ca_ channel inhibitor) or CbTX with IbTx.^35^ In addition, [Ca^2+^]_o_ stimulated NO production and whole‐cell IK_Ca_ channel currents in VECs, which were blocked by Calhex‐231. These findings indicate that the stimulation of the CaSR on VECs induces vasorelaxations via two endothelium‐dependent pathways, NO production, and activation of BK_Ca_ channels in VSMCs and stimulation of IK_Ca_ channels in VECs that induce endothelium‐derived hyperpolarization. In follow‐up studies using rabbit and WT and TRPC1 KO mouse mesenteric arteries, the stimulation of the CaSR by [Ca^2+^]_o_ was shown to induce vasorelaxations via the activation of a 6pS heteromeric TRPV4‐TRPC1 channel in VECs which likely led to Ca^2+^ influx that induced NO production but not IK_Ca_ channel activation.^60^, ^61^ Interestingly, a Gd^3+^‐sensitive process, possibly involving TRPC3 channels in VECs, was proposed to be involved in the CaSR‐mediated activation of IK_Ca_ channels.^61^

In rat mesenteric vascular beds, R‐568 produced dose‐dependent vasodilatations, which at low concentrations produced preceding vasoconstrictions in spontaneous hypertensive but not control animals.^62^ In addition, both vasodilatations and vasoconstrictions were blocked by the calcilytic Calhex‐231 and inhibition of eNOS (L‐NAME), COX (indomethacin), BK_Ca_, and IK_Ca_ channels (CbTx) and removal of a functional endothelium.

### 6.3 Vascular smooth muscle‐dependent actions

In Section 5, we discussed the importance of stimulation of the CaSR on VSMCs in mediating vasoconstrictions (see work of ^26^, ^29^, ^32^). It is proposed that these CaSR‐induced vasoconstrictions are mediated by the activation of the G_q/11_ protein‐coupled signaling pathway involving PLC and IP_3_‐mediated increase in [Ca^2+^]_i_. In gerbil spiral modiolar arteries, [Ca^2+^]_o_‐induced constrictions were reduced by depleting internal Ca^2+^ stores with thapsgargin or ryanodine and inhibiting PLC with U73122 but not by its inactive analog U73343 or the VGCCs blocker nifedipine.^26^ In rat pulmonary artery, [Ca^2+^]_o_‐induced constrictions were also inhibited by U73122, thapsgargin, and the nonselective IP_3_ receptor inhibitor 2‐APB.^29^

## 7 ROLE OF THE CaSR IN PROLIFERATIONS AND CALCIFICATION OF VSMCS

Proliferation and calcification of VSMCs are associated with vessel remodeling, changes in contractility, and cardiovascular disease, and there is substantial evidence for a role for the CaSR in these processes.

In rat aortic SMCs, increasing [Ca^2+^]_o_ augmented DNA synthesis and the number of VSMCs which were blocked by the calcilytic NPS2390 but not by the expression of a dominant negative CaSR, indicating a possible contribution from the CaSR and mGluR.^28^ However, in human aortic SMCs, the calcimimetic neomycin induced cell proliferation through PLC, IP_3,_ and MAP Kinase‐mediated pathways which were reduced by the knockdown of the CaSR by siRNA.^63^ Furthermore, rat thoracic aorta SMCs from spontaneous hypertensive rats (SHRs) had increased CaSR expression compared to normotensive animals and Calhex 231 reduced this increased CaSR expression and also reduced cell cycle markers and cell proliferation.^34^ These studies suggest that the stimulation of the CaSR is involved in increasing the proliferation of VSMCs.

In contrast, there have also been several studies that indicate the stimulation of the CaSR may reduce the proliferation of VSMCs. In A7r5 VSMCs, the calcimimetic calindol and increasing [Ca^2+^]_o_ reduced cell proliferation induced by high glucose and H_2_S.^64^ Moreover, Qu et al. ^33^ showed in thoracic aorta SMCs from SHRs had reduced CaSR expression and increased cell proliferation (opposite to findings of ^34^). In addition, Sun et al. ^65^ showed that R‐568 reduced aortic remodeling in SHRs, whereas Zhao et al. ^66^ demonstrated that NPS‐2143 increased the proliferation of rat thoracic aorta SMCs from SHR compared to normotensive rats. These studies also indicated an association between the stimulation of the CaSR having an anti‐proliferation action on VSMCs and a decrease in the renin‐angiotensin‐aldosterone system (RAAS).^65^, ^66^ It is important that further work is carried out on the CaSR and proliferation of VSMCs as although current available data may be variable, it does highlight the potential for CaSR therapeutics, whether calcimimetics and/or calcilytics, to reduce proliferation associated with cardiovascular disease.

The deposition of Ca^2+^ into VSMCs leading to their calcification has a profound effect of vascular function, including changes in wall stiffness, contractility, and development of atherosclerosis. The CaSR has been shown to be a likely important regulator of this calcification process. In rat and bovine aortic SMCs, increased [Ca^2+^]_o_ and the calcimimetics neomycin and AMG‐641 enhanced the expression of the extracellular Ca^2+^ binding protein matrix G1a (MGP) to reduce the calcification of vessels.^67^, ^68^ In addition, in rat VSMCs, the calcimimetic calindol inhibited high Pi‐induced calcification and increased MGP expression.^69^

Alam et al. ^31^ demonstrated in human tibial arteries that CaSR expression was reduced in calcified arteries. Moreover, they showed in bovine VSMCs that CaSR expression was reduced using culture conditions with a mineralization medium, which was enhanced by the expression of a dominant‐negative CaSR (arginine to glutamine mutation [R185Q]) and reduced by the calcimimetic R‐568. Ivanovski et al. ^70^ also illustrated that R‐568 could delay the calcification of the aorta in ApoE^−/−^ mice and that in cultured human aortic SMCs and the R‐568‐induced reduction of high mineral‐mediated calcification which was inhibited by CaSR siRNA. In cultured human aorta VSMCs harvested following bypass surgery, application of high [Ca^2+^]_o_, R‐568 and AMG641 increased CaSR expression and also increased mineral and collagen matrix secretion and reduced calcification that were all reduced following treatment with CaSR siRNA.

In a transgenic mouse study, Schepelmann et al. ^5^ showed that although there was not a significant increase in calcification of vessels from VSM‐specific CaSR KO mice, there was a mineral imbalance with hypercalcemia, hypercalciuria, hyperphosphaturia, and osteopenia, and cultured VSMCs from these KO mice calcified faster in the presence of mineralization conditions. Importantly, this study reported that the function of the CaSR in parathyroid and kidneys was unaffected in this KO mice.

Together, these studies indicate that a reduction in expression and activity of the CaSR is associated with calcification of blood vessels and that calcimimetics may be a potential target for reducing this calcification. Moreover, the CaSR is likely to be an important role in total body mineral ion homeostasis, and as such, the vasculature might be considered a calciotropic tissue.

## 8 ROLE OF THE CaSR IN REGULATING SYSTEMIC BLOOD PRESSURE AND PULMONARY ARTERY

This review provides evidence that the CaSR is involved in regulating vascular contractility, but a critical question is whether the CaSR has an effect on systemic and pulmonary blood pressure?

### 8.1 Systemic blood pressure

#### 8.1.1 In vivo animal studies

In animal studies, the calcimimetic R‐568 induced similar reductions in blood pressure in nephrectomized compared to parathyroidectomy rats^71^ and decreased blood pressure in SHRs but only when parathyroid glands were present.^72^, ^73^ In contrast, the calcilytic NPS 2143 increased blood pressure in rats which was prevented by removing parathyroid glands ^74^ and pretreatment with voltage‐gated Ca2+ channels (VGCC) and angiotensin receptor blockers.^75^ In uremic and nonuremic rats, cinacalcet produced an acute increase in blood pressure associated with changes in vascular bed resistance indicating that stimulation of the CaSR may be involved in both vasoconstriction and vasodilatation.^76^ Treatment with R‐568 for 8 weeks reduced the increased blood pressure and reversed the lower expression of the CaSR present in SHRs.^65^

In patients with persistent hyperparathyroidism following renal transplant, the calcimimetic cinacalcet was shown to reduce associated hypertension indicating its suitability for controlling blood pressure.^77^, ^78^

These findings indicate potential vasculature actions of the CaSR in regulating blood pressure. However, caution is needed when interpreting these studies since the CaSR may also regulate blood pressure via reducing renin levels,^79^ altering renal tubule Ca^2+^‐reabsorption,^80^ and controlling parathyroid gland‐mediated secretions of PTH and parathyroid hypertensive factors which are known to regulate blood pressure.^81^

#### 8.1.2 Human studies

There have been several clinical trials investigating the effect of the calcimimetic cinacalcet in reducing cardiovascular disease in patients with chronic kidney diseases, secondary hyperparathyroidism, and undergoing hemodialysis (see review by ^82^). The proposed hypothesis of these studies was that cinacalcet is likely to reduce cardiovascular events through, in part, reducing calcification of the vasculature. In an observational study, Block et al. ^83^ showed that cinacalcet prescribed with vitamin D produced a reduction in all‐cause and cardiovascular disease mortality. In the ADVANCE (A randomized study to evaluate the effects of cinacalcet plus low‐dose vitamin D on vascular calcification in subjects with chronic kidney disease receiving haemodialysis) trial, Raggi et al. ^84^ investigated over 300 patients given vitamin D sterols with and without cinacalcet and showed that calcification scores recorded after one year trended toward a reduction in the aorta and mitral valve and were significant for the aortic valve. However, in the EVOLVE (Evaluation of cinacalcet HCl therapy to lower cardiovascular events) trial, Chertow et al. ^85^ showed in a multicentered and multicountry study of nearly 4000 patients that cinacalcet in combination with vitamin D did not reduce time to death and nonfatal cardiovascular events. In all these studies, that there was no change in systemic blood pressure in cinacalcet patient groups.

#### 8.1.3 Role of CaSR in pulmonary artery hypertension (PAH)

PAH is associated with a rise in vascular resistance caused by vessel remodeling involving proliferation and increased contractility. A role for the CaSR in these processes and development of PAH has been widely studied and probably represents the most comprehensive evidence available that the CaSR is a potential therapeutic target to treat a cardiovascular disease. As such, there is a substantial literature on the role of the CaSR in the pulmonary vasculature and PAH which we highlight in brief here (see reviews by ^86^, ^87^, ^88^ for details).

In initial studies, a rise in [Ca^2+^]_o_ and hypoxia was shown to increase [Ca^2+^]_i_ levels, proliferation of pulmonary artery SMCs, and contraction of pulmonary arteries, which were blocked by calcilytics and CaSR knockdown.^29^, ^89^, ^90^ Yamamura et al. ^91^ showed that PASMCs from idiopathic PAH (iPAH) patients had increased CaSR expression, increased [Ca^2+^]_i_, and proliferation to R‐568 which was reduced by CaSR siRNA. Yamamura et al. ^91^ also showed that CaSR expression was increased in monocrotaline (MCT)‐induced PAH rats and that the calcilytic NPS 2143 reduced pulmonary artery pressure and proliferation of PASMCs from these animals. In addition, they showed that in hypoxia‐induced PAH mice, increased pulmonary artery pressures were reduced by NPS 2143. The cellular mechanisms coupled to CaSR‐induced PAH are unclear, but it has been proposed to involve the canonical TRP 6 (TRPC6), protein kinase B phosphorylation, and Notch signaling.^92^, ^93^ Moreover, a recent study indicates that several CaSR variants located in exon 7 are associated with PAH.^94^

In an interesting study, Yamamura et al. ^95^ proposed that the dihydropyridine (DHP) class of VGCC blockers, but not the nondihydropyridine class, activated the CaSR in PASMCs from iPAH patients and enhanced PAH in MCT‐induced PAH rats. These unique findings suggested that DHP drugs act as potential positive modulators of the CaSR and proposed that the treatment of PAH with these drugs may make symptoms worst.

## 9 SELECTIVITY OF CALCIMIMETICS AND CALCILYTICS IN VASCULAR FUNCTION STUDIES

Understandably, many studies investigating the functional expression of the CaSR on the vascular function use calcilytics and calcimimetics to infer that the effects of increasing [Ca^2+^]_o_, the physiological activator, are mediated by the CaSR. As such, the selectivity of these agents in the vasculature is critical for appropriate interpretation. There is evidence that in the vasculature, both calcilytics and calcimimetics have CaSR‐independent actions, including acting as VGCC blockers. This is important since the VGCC‐mediated Ca^2+^ influx is pivotal for many vascular functions including contractility (e.g., vasoconstrictor‐mediated precontracted tone and regulation of vascular bed resistance as a significant VGCC‐dependent component).

The concentration of calcilytics and calcimimetics used is paramount when distinguishing between CaSR‐dependent and possible CaSR‐independent actions in the vascular function. These agents act within the nanomolar range to modulate the CaSR at parathyroid glands and kidneys to regulate Ca^2+^ homeostasis, whereas off‐target actions of these agents in the vasculature are reported at micromolar concentrations.

In HUVECs and HAECs, the calcimimetic R‐568 and its enantiomer S‐568 at concentration between 1 and 100 μM increased [Ca^2+^]_i_ and NO production which were not blocked by Calhex‐231.^37^ Moreover, high concentrations of R‐568 and S‐568 (about 70 μM) produced reductions in systemic blood pressure, increased heart rate, and increased blood flow.^96^ As S‐568 is a far less potent calcimimetic than R‐568 at the CaSR and these cardiovascular actions occurred at concentrations 3000 times higher that plasma levels required to alter PTH levels, it was proposed that R‐568 and S‐568 were acting via CaSR‐independent mechanisms, possibly at VGCCs in the vasculature.

In rat mesenteric arteries, the calcimimetics calindol and cinacelcet at micromolar concentrations inhibited precontracted tone by methoxamine, KCl, and the VGCC activator BAY8644 which were not prevented by Calhex‐231,^48^ indicating a likely direct blocking action of these agents on VGCCs to reduce contractility. In rabbit mesenteric arteries, MO‐ and KCl‐induced precontracted tone and contractions induced by Ca^2+^ influx through VGCCs were inhibited by micromolar concentrations of Calhex‐231 and NPS 2143. In gold standard experiments, Calhex‐231, NPS 2143, and calindol inhibited VGCCs using whole‐cell patch clamp techniques and Ba^2+^ as the charge carrier, providing definitive evidence that commonly used calcilytics and calcimimetics block these channels in VSMCs.^97^

In addition, VGCC‐mediated pathways are thought to be involved in renin release,^98^ and therefore the use of higher concentrations of calcilytics and calcimimetics in *in vivo* studies may modulate blood pressure by regulating renin release and the RAAS.

These reports indicate that caution is needed when using calcimimetics and calcilytics in studies to infer the effect of stimulating the CaSR in the vasculature, especially at micromolar levels and possibly high nanomolar levels, where these agents may act via the CaSR and also inhibit VGCCs. This should perhaps be not entirely unexpected as Calhex‐231, NPS 2143, and calindol are structurally related to the phenylakylamines groups of VGCCs, with fenidiline, the lead compound, used to develop the calcimimtics R‐568, cinacalcet, and calindol, a potent VGCCs blocker.^99^, ^100^, ^101^, ^102^, ^103^ In addition, more recently developed calcilytics, AXT914 and AFT936,^13^ also reduce methoxamine‐induced precontracted tone in rat and mice mesenteric arteries at micromolar concentrations (authors unpublished data).

## 10 FUTURE THOUGHTS

This commentary clearly shows substantial evidence for the functional expression of the CaSR in the vascular tissue and that the activation of this receptor has profound effects on the vascular function including contractility and proliferation and calcification of VSMCs. From the number of studies by different research groups using differing vasculature beds from different animal species and human tissues, the nature of these actions of the CaSR on the vascular function is likely to be canonical. As such, investigating the effects of CaSR on the vasculature is likely to increase our fundamental knowledge of vascular function and potentially discover new therapeutic targets for vascular diseases.

Although the importance of the CaSR in vascular contractility is clearly linked, there is also much complexity surrounding its diverse actions. This is somewhat inevitable considering the functional expression of the CaSR in multiple cell types, for example, perivascular sensory nerves, VECs, and VSMCs, which couple to multiple cellular signaling pathways to induce both vasodilatations and vasoconstrictions. How can we make more sense of these diverse findings in future studies? What are potential important questions to ask? We certainly need a more detailed picture of the effect of the CaSR on vascular contractility in the human tissue to frame results from animal studies. Moreover, a more systematic approach to using animal studies might be useful. Evidence highlighted in this review article indicates that the CaSR in larger, more conduit functioning vessels, such as the aorta and pulmonary arteries, may be involved in regulating vasoconstriction through CaSR‐mediated pathways in VSMCs, whereas the activation of the CaSR in resistance vessels, for example, mesenteric arteries, is involved in regulating vasodilatation through CaSR‐mediated pathways in perivascular sensory nerves and VECs. Expanding these ideas would be useful, perhaps using more molecular approaches, such as further studies using the smooth muscle cell‐specific CaSR KO mouse and developing a sensory nerve and endothelium‐specific CaSR KO mice models.

Although there is increasing evidence that the CaSR has an important role in pulmonary hypertension (see above and reviews by ^86^, ^87^, ^88^), there is limited information on the role of CaSR in regulating systemic hypertension. This would be greatly advanced with detailed studies using hypertensive animal models such as SHRs and Ang II‐induced hypertensive mice and hypotensive models such as LPS‐induced sepsis.

One of the main restrictions when studying and interpreting the action of the CaSR in the vasculature, whether using in vitro or in vivo approaches, is concentrations of calcilytics and calcimimetics used which is often far greater than the concentrations known to be required to affect the action of the CaSR at parathyroid glands and kidney. At higher concentrations, the clear inhibitory action at VGCCs in VSMCs is a significant issue when interpretating these results on the vascular function. As such, lower concentrations of calcilytics and calcimimetics need to be used in vascular function studies, for example, wire myography. Perhaps the use of lower concentrations of vasoconstrictors to generate precontracted tone may help, as is the use of pressure myography.

## 11 SUMMARY

There is clear evidence that the stimulation of the CaSR has important roles in regulating the vascular function through the expression of the receptor on perivascular sensory nerves, VECs, and VSMCs which are associated to multiple signaling pathways that induce both vasoconstrictions and vasodilatations, proliferation, and calcification of VSMCs. As such, the CaSR is likely to be a potential novel therapeutic target for vascular diseases.

## AUTHOR CONTRIBUTIONS

A.P.A and H.Z.E.G wrote the manuscript and agreed to the final submitted article.

## CONFLICT OF INTEREST STATEMENT

The authors declare no conflicts of interest.

## ETHICS STATEMENT

All animal procedures were carried out in accordance with guidelines laid down by City St. George's, University of London Animal Welfare Committee and conform with the principles and regulations described by the Service Project License: 70/8512.

## Data Availability

The data that support the findings of this study are available from the corresponding author upon reasonable request.
